# Community-Based Surveillance of Acute Flaccid Paralysis: A Review on Detection and Reporting Strategy

**DOI:** 10.1007/s44197-025-00349-2

**Published:** 2025-02-20

**Authors:** Gelane Biru, Honey Gemechu, Eyerusalem Gebremeskel, Hundessa Daba Nemomssa, Kokeb Dese, Efrem Wakjira, Gashaw Demlew, Dessalew Yohannes, Ketema Lemma Abdi, Hamdia Murad, Elbetel Taye Zewde, Bontu Habtamu, Mesfin Tefera, Mikias Alayu, Netsanet Workneh Gidi, Filimona Bisrat, Tenager Tadesse, Legesse Kidanne, Se-woon Choe, Jude Kong, Gelan Ayana

**Affiliations:** 1 School of Biomedical Engineering, Jimma Institute of Technology, Jimma University, 378 Jimma, Ethiopia; 2 Faculty of Civil and Environmental Engineering, Jimma Institute of Technology, Jimma University, 378 Jimma, Ethiopia; 3 Faculty of Computing and Informatics, Jimma Institute of Technology, Jimma University, 378 Jimma, Ethiopia; 4 Faculty of Public Health, Department of Reproductive Health, Jimma University, 378 Jimma, Ethiopia; 5 Computer Vision Division, Ethiopian Artificial Intelligence Institute, 40782 Addis Ababa, Ethiopia; 6 Center for Public Health Emergency Management (PHEM), Ethiopian Public Health Institute (EPHI), Addis Ababa, Ethiopia; 7 Department of Pediatrics and Child Health, Jimma Institute of Health, Jimma University, 378 Jimma, Ethiopia; 8 CORE Group Partners Project, 5674 Addis Ababa, Ethiopia; 9 Department of IT Convergence Engineering, Kumoh National Institute of Technology, Gumi, 39253 Korea; 10 Artificial Intelligence and Mathematical Modeling Lab (AIMMLab), Dalla Lana School of Public Health, University of Toronto, 155 College St Room 500, Toronto, ON M5T 3M7 Canada; 11 Department of Mathematics, University of Toronto, Bahen Centre for Information Technology, Room 6291, 40 St. George Street, Toronto, ON M5S 2E4 Canada; 12 Africa-Canada Artificial Intelligence and Data Innovation Consortium (ACADIC), Toronto, Canada; 13 Global South Artificial Intelligence for Pandemic and Epidemic Preparedness and Response Network (AI4PEP), Toronto, Canada

**Keywords:** Polio, Acute flaccid paralysis, Community-based surveillance, Active surveillance

## Abstract

Polio is a highly contagious viral disease that primarily affects children under 15, often leading to permanent paralysis, known as acute flaccid paralysis (AFP). AFP surveillance is essential for the eradication of polio, with community-based surveillance (CBS) playing a pivotal role in detecting and reporting cases. CBS improves the timeliness and accuracy of AFP detection, but challenges such as underreporting, delays, and low community awareness persist. Strategies involving use of mobile applications, awareness campaigns, and improvements in healthcare infrastructure were implemented to improve CBS of AFP. While numerous case studies from various countries illustrate the implementation of CBS, a comprehensive synthesis of these studies across diverse contexts is limited. This paper examines state-of-the-art CBS approaches for AFP, analyzing progress, challenges, and potential solutions. A targeted literature review of English-language studies published between 2004 and 2024 was conducted, focusing on the roles of communities, technological integration, and practical recommendations, while excluding studies that lacked methodological rigor or direct relevance. The review revealed that CBS has significantly advanced the global fight against polio by increasing community awareness, enabling earlier detection, and improving the reporting of AFP cases. However, issues such as security concerns, delayed reporting, low levels of community awareness, and underutilization of technology persist. This review recommends strengthening organizational structures, improving healthcare access, raising community awareness, and using technology for more efficient AFP surveillance. The implication of this work is beyond polio as it offers a comprehensive framework for integrating disease surveillance, technology and community involvement to strengthen public health strategies and build robust health systems.

## Introduction

Polio is a highly contagious viral disease caused by a poliovirus that belongs to the family Picornaviridae [1–3]. It causes a sudden onset of muscle weakness or floppiness and ends with permanent or irreversible disability of movement. The paralysis occurs in 1 out of every 200 people infected, and 5–10% of them die when their breathing muscles unable to function [4, 5]. Despite these, 90% of cases are asymptomatic and the remaining cases manifest as aseptic meningitis, which is self-limiting condition characterized by fever, headaches, sore throats, malaise and myalgia [1, 6]. The disease spread to any vulnerable community around the world and infects anyone at any age [7]. The majority of the cases happen in those under the age of five, but all children under the age of fifteen are susceptible [8, 9].

In 1988, the World Health Assembly passed a resolution urging the eradication of polio worldwide, sparking the beginning of the Global Polio Eradication Initiative (GPEI) [10, 11]. Following that, national governments, World Health Organization (WHO), Rotary International, the Vaccine Alliance, Gavi, the Bill & Melinda Gates Foundation, and the US Centers for Disease Control and Prevention (CDC) joined the initiative [11, 12]. Since then, a lot has been improved regarding polio eradication. For instance, in 1988 there was an estimated 350,000 cases of wild poliovirus (WPV) types 1, 2 and 3 in over 125 endemic countries. Out of the estimated values, the WPV type 1 decreased to hundreds of reported cases in Afghanistan and Pakistan in 2019 [13]. The last WPV type 2 was reported in 1999, while WPV type 3 was eliminated in 2020 out of the three strains of the virus (type 1, type 2, and type 3) [11, 14, 15]. In 2023, there were 12 cases of reported WPV type 1 in both Afghanistan and Pakistan, showing a decrement when compared to the 22 cases in 2022 [16]. There was also a decrement in number of reported polio cases caused by circulating vaccine derived poliovirus (cVDPV). In 2023, Eight countries reported new cases of cVDPV, and 24 countries continued to have outbreaks of the virus since 2022 [16]. Until recently, two nations, Afghanistan and Pakistan, are left with endemic WPV type 1 [16, 17]. As we write this paper in July 2024, there is a news reported from Gaza about new outbreak of WPV type 2 [18]. The WHO believes that if this fresh outbreak is not contained as soon as possible and as effectively as possible, there is a significant chance that the virus would spread from Gaza to other nations [19, 20].

Surveillance is the cornerstone for eliminating polio, halting the virus's spread, and identifying its transmission [16, 21]. Polio surveillance tasks encompass thorough inspections of each residence, monitoring any changes in health trends, overseeing illness prevention initiatives, and managing reactions to outbreaks. There are two different kinds of polio surveillance: Environmental surveillance (ES) and acute flaccid paralysis (AFP). The prior, ES, refers to a systematic sewage sample collecting and testing that used to determine the presence and spread of the virus [16, 22–24]. The specimens are obtained from different sites including source of water, sewage lines, or probably areas where vectors are found [25]. The later, that uses an active intervention method known as AFP surveillance, is the key to detecting the spread of WPV [26]. The AFP surveillance process includes both health facility and community-based approach to identify and monitor poliovirus (PV) circulation [27]. Health facility-based surveillance involves the use of health workers to identify, report and manage any AFP case that the health facilities come across in their normal practice, collect stool samples from patients and manage the data [28–30]. Community-based surveillance (CBS), on the other hand, leverages community volunteers (CVs) and local leaders to identify AFP cases through active case screening. This process involves detecting, reporting, investigating, and initiating response actions, ensuring that even hard-to-reach communities are effectively monitored and addressed [31, 32]. Both the health facility and CBS approaches pass information to each other, undertake joint training and ensure that their actions overlap in a bid to ensure eradication of PV [33].

AFP surveillance has a well-established structure from national level down to the community [34]. Despite its effectiveness, AFP surveillance efforts encounter numerous challenges. Factors such as inadequate healthcare infrastructure [35], limited-service accessibility [36], deficient transportation and communication [37, 38], geographic inaccessibility [32], high population mobility [32], spread of emergency infectious disease, security concerns [32, 39, 40], cultural and language barriers, and low community awareness contribute to underreporting and delayed detection of AFP [2, 41]. To address these challenges, initiatives have been undertaken, including the improvement of healthcare infrastructure, heightened community awareness, and the deployment of mobile clinics and community health workers. Moreover, the use of digital tools, such as auto-visual AFP detection and reporting (AVADAR) system [42] and open data kit (ODK) [43], along with deep learning methods for identifying AFP cases from images [44] are among the key innovations that enhanced the effectiveness of CBS for AFP detection and reporting.

**Fig. 1:**
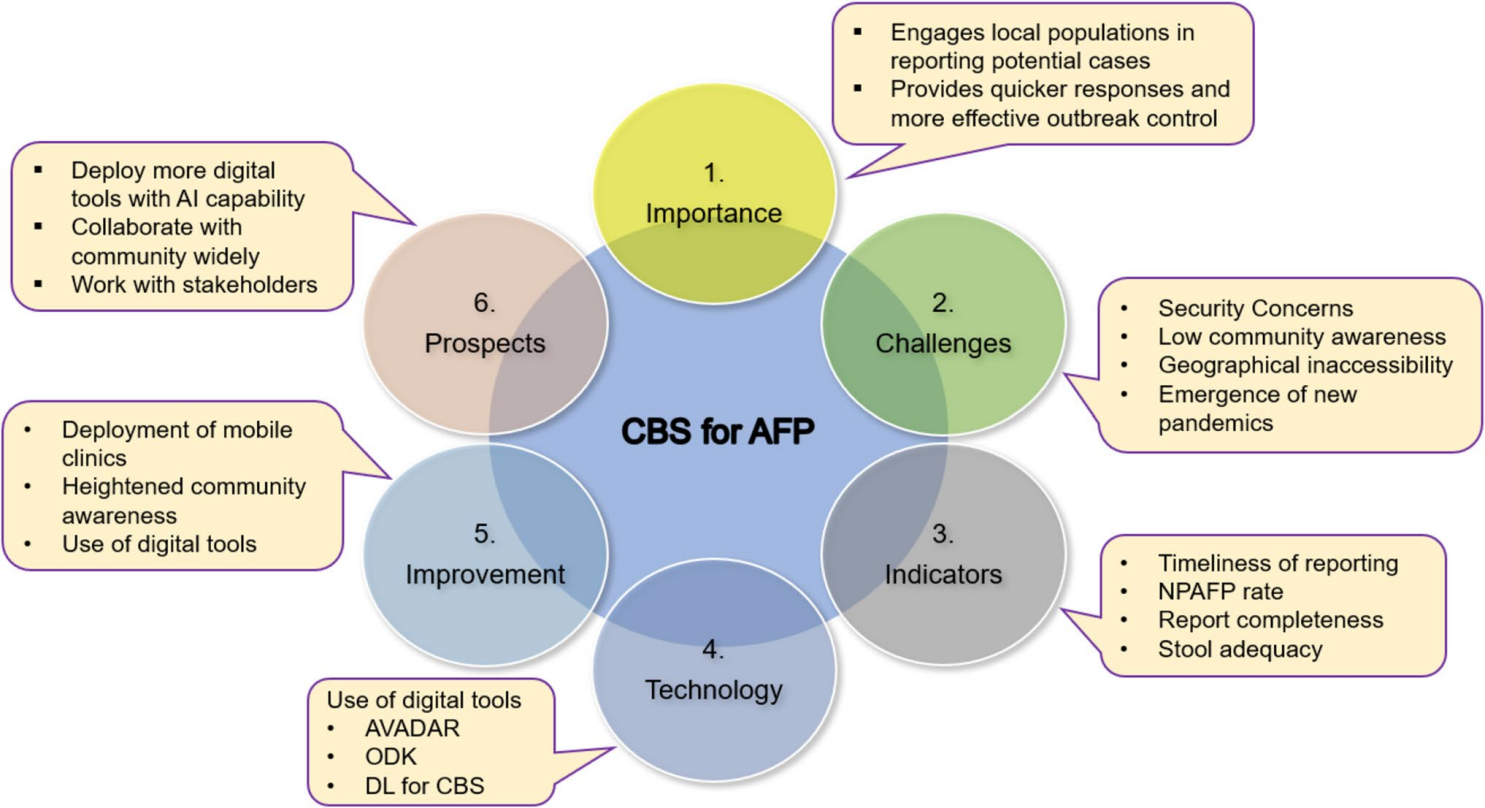
Summary of community-based acute flaccid paralysis surveillance as a key strategy for timely detecting and reporting cases. *AFP* acute flaccid paralysis, *AVADAR* auto-visual acute flaccid paralysis detection and reporting, *CBS* community-based surveillance, *DL* deep learning, *ODK* open data kit

Several case studies from various countries that have implemented CBS for AFP, capturing the system's essence in different cultural, geographic, and socio-economic contexts, are available [33, 45–47]. However, comprehensive papers that synthesize and compare these experiences across diverse scenarios are rarely available. Most case studies focus on specific settings, highlighting localized challenges, innovations, and outcomes without providing a broader view of common patterns, lessons learned, and universal insights. A synthesis of these studies could offer valuable guidance for optimizing CBS of AFP globally, identifying best practices, adapting strategies to different environments, and addressing shared challenges more effectively. Figure 1 provides an overview of the general idea of community-based surveillance for acute flaccid paralysis.

Therefore, this paper aims to highlight the latest advancements and state-of-the-art approaches in CBS of AFP. By analyzing and synthesizing recent progress, the paper provides a comprehensive overview of how CBS systems have evolved, the improvements they have driven in AFP detection and response, and explores the innovative tools and strategies currently being employed. The paper also examines the persistent challenges that continue to hinder the full potential of CBS in various settings, such as issues related to community engagement, data accuracy, technological integration, and logistical barriers, especially in hard-to-reach areas. Furthermore, this work proposes potential solutions and actionable recommendations to address these challenges, offering insights that can be adapted to different contexts and contribute to the enhancement of global AFP surveillance efforts.

This paper makes a unique contribution as the first comprehensive review explicitly focused on CBS for AFP to the authors’ knowledge as no prior review has addressed this critical topic. While existing literature often emphasize localized case studies or isolated challenges in specific implementation contexts, this review stands out by synthesizing findings from diverse geographical and socio-economic settings, offering a broader perspective on CBS for AFP. Additionally, it highlights cutting-edge strategies and technological advancements, such as mobile health applications and AI tools for real-time data reporting and analysis, which enhance CBS efficacy. The review also addresses persistent issues in CBS, including community engagement and underreporting, and proposes practical solutions to overcome these challenges. Finally, the paper extends the implications of CBS beyond polio surveillance, suggesting that the insights gained can inform strategies for addressing other infectious diseases and contribute to a more comprehensive understanding of community health initiatives.

In general, this review is pivotal in shaping the future of CBS for AFP, offering innovative solutions that can significantly boost global polio eradication efforts. By enhancing early detection and reporting accuracy, it empowers communities to play a critical role in surveillance. The insights provided have the potential to transform policy and inspire new, multisectoral strategies that can effectively close the gaps in AFP surveillance and accelerate the end of polio worldwide.

## Methodology

This study employed a targeted literature search, to find the most relevant research on CBS for AFP in several databases, such as Google Scholar, PubMed, Scopus, Web of Science, ScienceDirect, JSTOR, DOAJ, IEEE Xplore, and ResearchGate. To refine the search results, the methodology used targeted keyword and phrase matching including polio, poliomyelitis, CBS, disease surveillance, AFP, and digital tools. The search was limited to articles published in English over the past 20 years (2004–2024) following the implementation of CBS in many epidemic countries to strengthen AFP surveillance. This approach ensured that the review included the most relevant and up-to-date findings reported on the topic.

Selection criteria were carefully designed to include the most impactful and relevant studies, prioritizing those directly addressing the role of communities in AFP surveillance activities. This focus aligns closely with the core objective of this review. To provide a broader perspective, studies that were not specifically centered on AFP but offered valuable insights into the general principles and practices of CBS were also considered. During the intensive review process, abstracts, methodologies, challenges, and technological advancements highlighted in the literatures found in the databases accessed were carefully evaluated to identify studies that contribute significantly to enhancing CBS systems. Conversely, most studies that focused on AFP surveillance without integrating community roles were excluded to maintain the review’s alignment with its primary focus. Subjective opinions, non-empirical studies, and research lacking methodological rigor were also omitted to ensure the review’s credibility and relevance. This thorough selection process resulted in a robust collection of literature that includes diverse case studies, reports, and analyses representing a broad spectrum of economic and geographic contexts. In this way, this work identified studies that showcased creative approaches to community involvement, demonstrated proven impacts on surveillance outcomes, and highlighted best practices for effectively detecting and reporting AFP.

Relevant data from the selected articles were carefully retrieved, including study aims, techniques, major findings, highlighted challenges, and practical recommendations for enhancing CBS of AFP. A rigorous thematic analysis organized the data into key categories, including community engagement, technological integration, and barriers to successful surveillance. This process enabled the extraction of critical information, resulting in a meaningful outlook, actionable recommendations, and well-founded conclusions.

## Community Based Disease Surveillance Approach

**Fig. 2:**
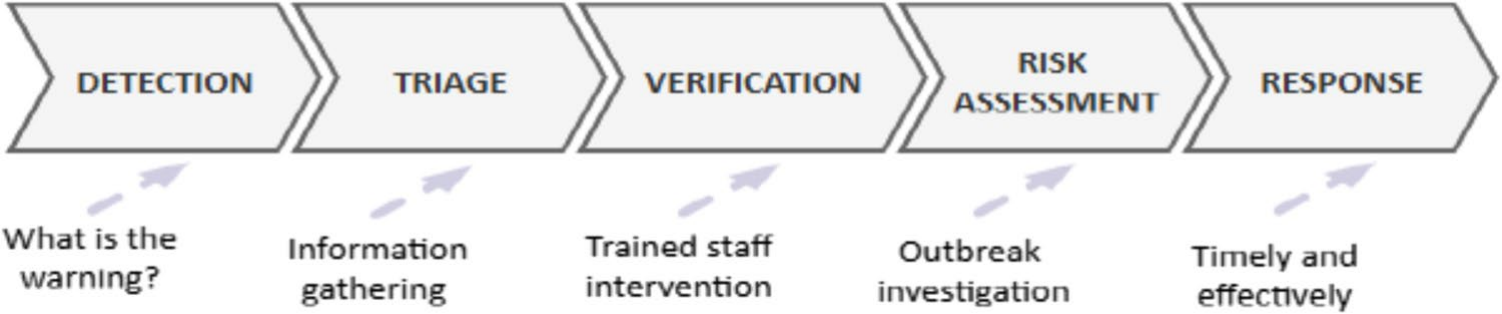
Community-based disease surveillance process

Disease surveillance relies on collecting, analyzing, and interpreting large volumes of data on diseases from various sources [48]. Traditionally, healthcare facilities collect this data, but in the case of infectious diseases, this approach may be ineffective, as it often happens too late. Communities are typically the first to detect when something new happens, making CBS a crucial component in early detection and response. CBS is a straightforward, flexible and reasonably priced public health program run by communities for the purpose of community protection [49, 50]. The first step in CBS is raising the community’s awareness on potential health hazards. The awareness creation program could take the form of closely monitoring an ongoing disease outbreak or an uncommon event that could point to a new health concern [51, 52]. To do CBS of diseases, a series of well-defined steps that guide the process from initial detection to an effective response is followed (see Fig. 2). This comprehensive CBS process includes key stages such as detection, triaging, verification, risk assessment, and ultimately, the coordinated response that involve different stakeholders at different levels.

Communities can significantly impact global health security, but only if they are equipped with the necessary means, resources, and involvement to identify threats, take preventive measures to halt pandemic or epidemic diseases, recognize potential illness occurrences, and promptly notify authorities to act [53]. This effectively expands the local surveillance reach and gives local ownership to the process [54, 55]. To achieve this, the community nominates influential and respected volunteers in each village, including traditional healers, educators, clergymen, birth attendants, leaders of women's groups, youth, and bonesetters [56, 57]. Thus, volunteers are selected based on some criteria that included diversity and representation of local subpopulation groups in terms of gender, language, literacy, recognition, ethnicity/tribe, and other categories.

**Fig. 3:**
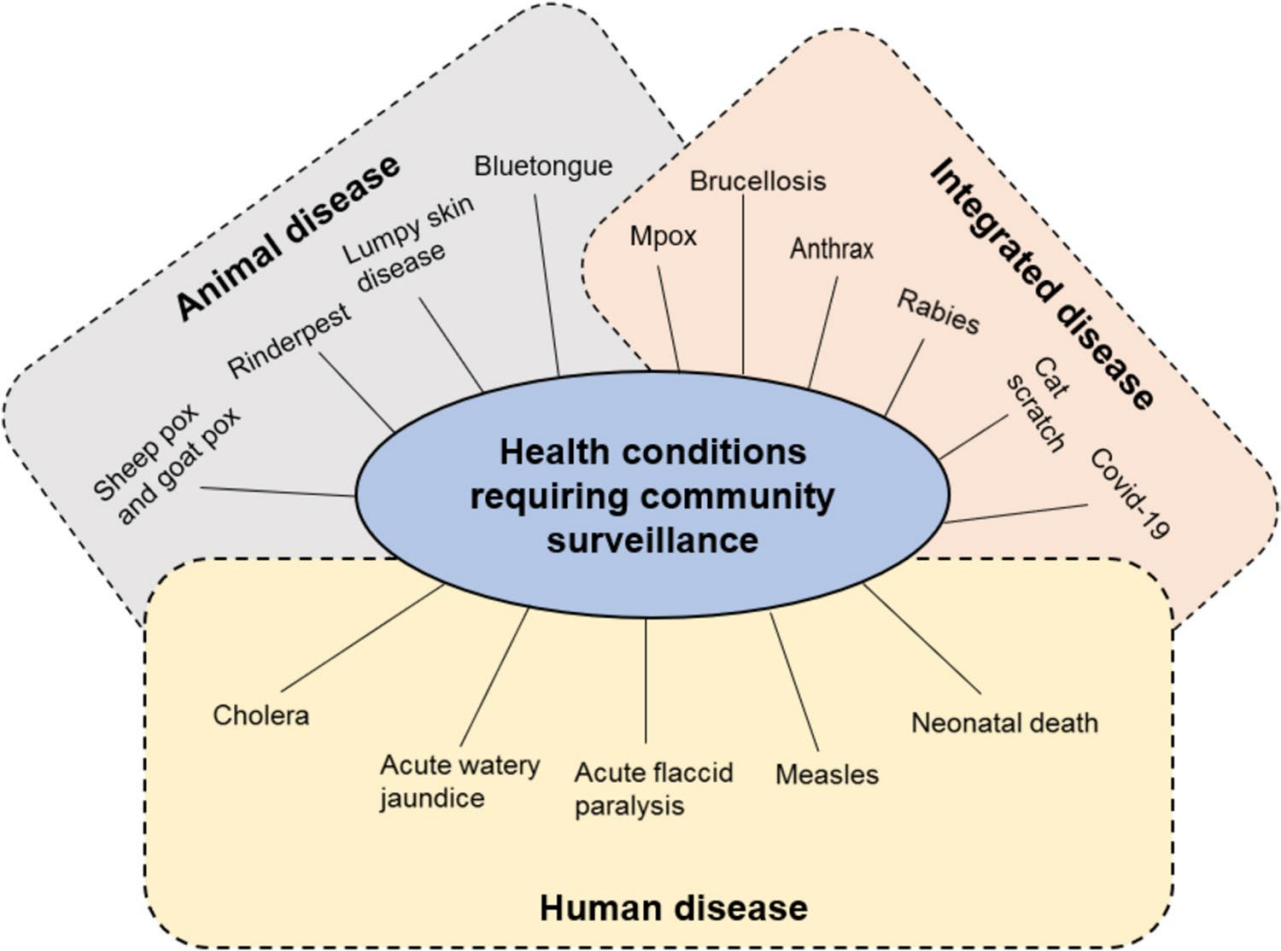
Animal, human and integrated diseases for which community-based surveillance is performed in many countries

Countries facilitate community-based disease surveillance through a variety of methods tailored to their specific contexts and needs. These approaches often include training local volunteers, leveraging traditional networks, and utilizing technology to enhance data collection and reporting. By adapting strategies to fit local conditions, countries monitor and respond to disease outbreaks within their communities. In some countries, CBS activities are facilitated by establishing various community groups, such as focus group discussions (FGDs), health development armies (HDAs), community health extension workers (HEWs), and key community informants (KCIs). These groups are responsible for notifying their respective healthcare focal persons about potential cases [50, 58]. Most of these community groups are trained to recognize diseases that require community surveillance. The assigned focal person is responsible for investigating cases, implementing response actions, and collaborating with the community. They ensure that the communities’ status is accurately reported based on the diseases case definitions. Additionally, a supervisor is needed to oversee volunteers within their designated catchment area, plan health-related activities, and double-check CBS alarms that volunteers raise. These diseases that are reported and monitored by the CBS are categorized into three groups based on their impact: those affecting humans, those affecting animals, and those that have an integrated impact on both, Fig. 3. In the case of human disease, only humans are susceptible to a disease; it cannot go from human to animal and vice versa. This also applies to animal diseases, which only affect animals. However, in the case of integrated disease, it might spread from animal to human, necessitating integrated surveillance, and communities are taught to identify them [59, 60]. As a result, informants are expected to be knowledgeable about all disease [61, 62].

## Acute Flaccid Paralysis

AFP is a clinical presentation of an individual with sudden weakness or paralysis of limbs or any part of the body [12, 63]. Flaccidity is the characteristic feature of AFP marked by the fact that the muscles do not have tonus. The distinctive feature of AFP is that the muscle weakness progresses very quickly and its development takes a few days to weeks, which differentiates it from other neurological diseases that cause gradual and chronic muscle weakness [64]. AFP may be caused due to different reasons that includes PV, Guillain-Barré syndrome (GBS), transverse myelitis, oncological disease, traumatic lesions and autoimmune disease. Out of these causes, GBS is the most common with occurrence rate of 50%. It is an autoimmune disease that affects the peripheral nervous system which results in the paralysis [45]. In the case of PV, it destroys anterior horn cells of the spinal cord and brain that results in upper and lower limb paralysis or both.

**Fig. 4:**
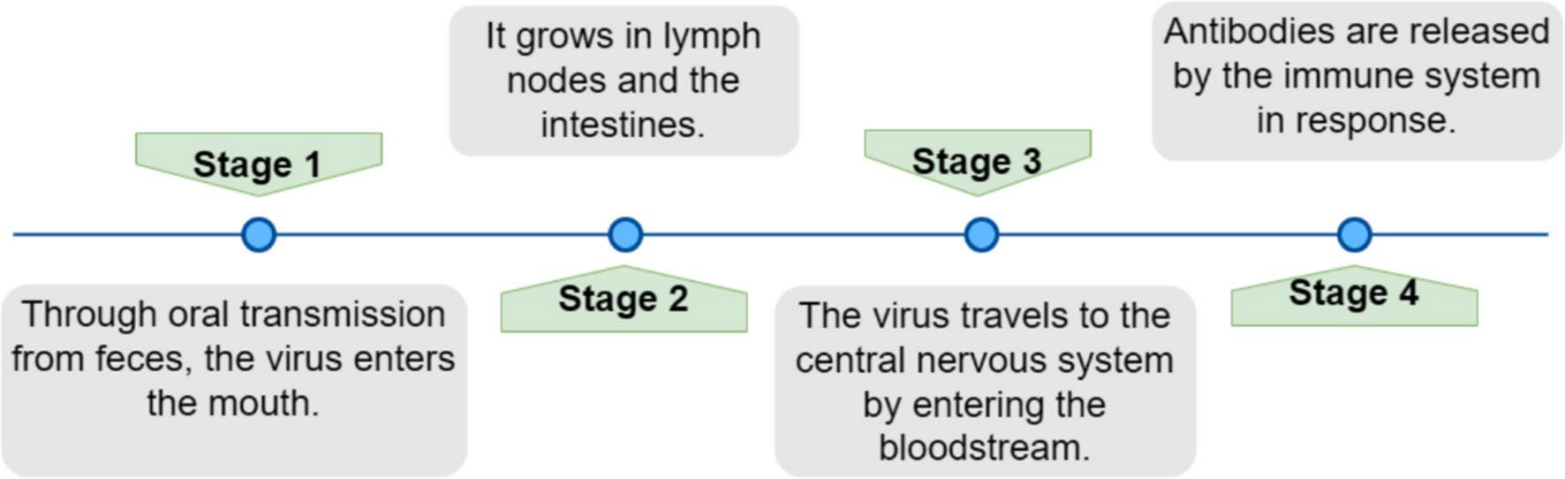
The stages how polio virus enters to human and its transmission phases

Poliovirus has a transmission mechanism that makes children under age of 15 more vulnerable to infections. The primary means of viral transmission is faecal-oral route, especially in areas with inadequate sanitation and hygiene [65, 66]. The virus enters through the mouth and then spreads down to pharynx. Figure 4 describes the stages of how PV enters and transmits in human. Polio needs accurate and early diagnosis as early as possible before it manifests as a paralysis but, since there is no cure for it, once it happens, supportive treatments, nutritional support and rehabilitation therapy are the only means to help the patient [67]. Early and accurate identification and reporting of virus carriers using the gold standard approach called AFP surveillance helps halt the transmission chain, stop the spread of the disease, and efficiently control outbreaks.

## Acute Flaccid Paralysis Surveillance

Since the WHO initiated the GPEI in 1988, AFP surveillance has been an important public health initiative in many countries [68, 69]. GPEI commenced a four-part eradication approach. The approach includes, administering supplemental doses of oral polio vaccine (OPV) or under five-years old children on national immunization days (NIDs), a localized mopping up vaccination campaigns, routine immunization (RI) of infants under one year old with at least three doses of OPV, and a sensitive surveillance system [70]. Importantly, the global efforts set out a high-quality surveillance of AFP as a gold standard for entire polio eradication [38, 70].

**Fig. 5:**
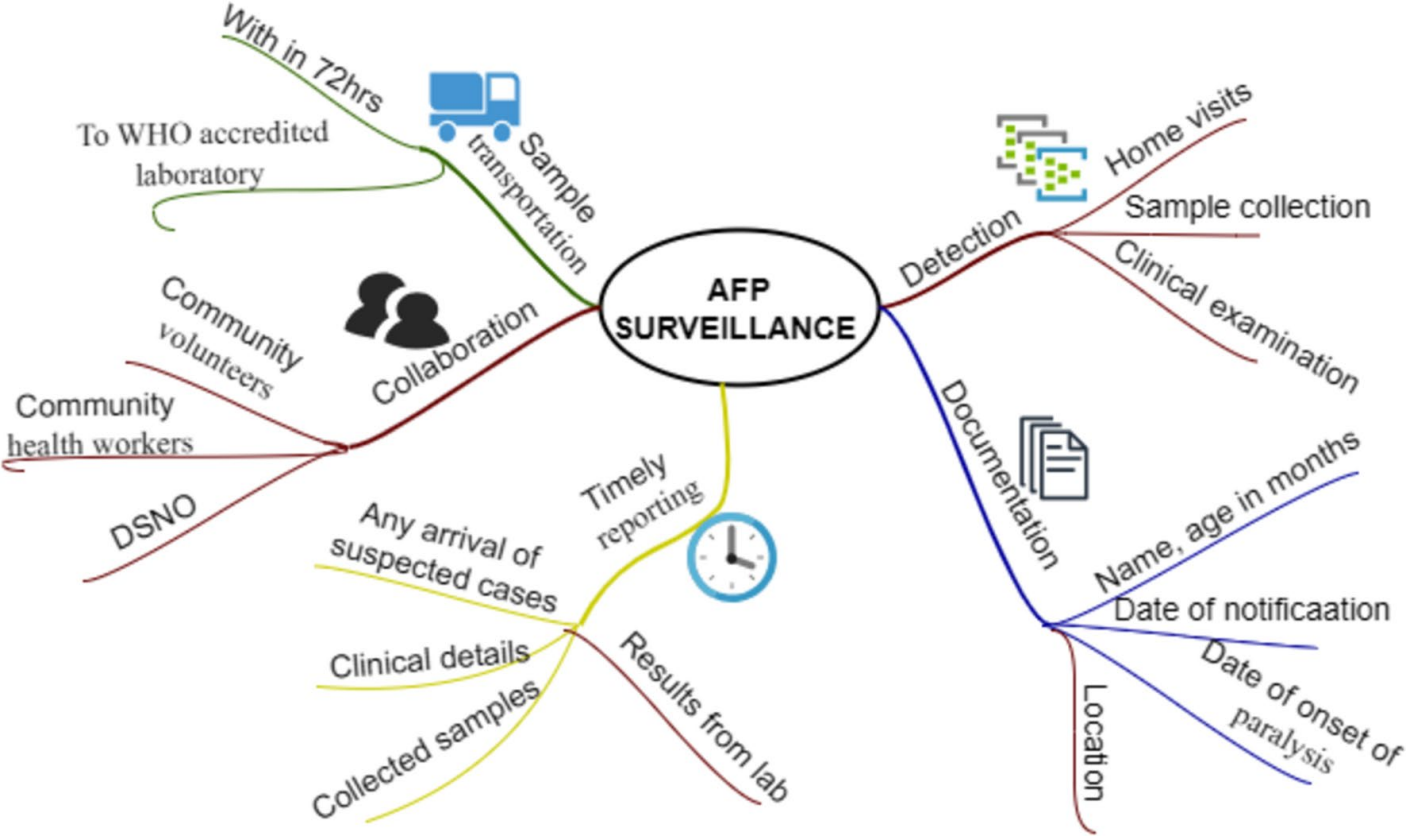
Acute flaccid paralysis surveillance system components and activities performed. AFP, acute flaccid paralysis; DSNO, disease surveillance and notification officer

The AFP surveillance activities are primarily locating and notifying parents of children with AFP signs, then transporting stool samples from the children for examination, isolating and characterizing the PV in the laboratory, and lastly mapping the virus to ascertain the strain's origin [71, 72] (see Fig. 5). In fact, surveillance of AFP is challenging because of the majority of polio cases do not result in paralysis [22, 73]. That is why an AFP surveillance system needs to be able to identify two cases of AFP for every 100,000 children < 15 years in order to be considered effective [74].

The AFP surveillance encompasses detection of enterovirus from stool of children under the age of 15 years [26]. The stool samples from each detected case of AFP must be collected and transferred by reverse cold-chain transfer to a laboratory approved by the WHO in order to identify the polio enterovirus. Once the paralysis starts, two stool samples are collected within 14 days, 24 h apart. The acquired samples are received by the laboratory in 72 h following the first sample collection. Processing the stool samples and investigating the cause of each case is then considered as appropriate in AFP surveillance activities [46, 75, 76].

To achieve high-quality surveillance that can quickly and efficiently identify, gather and report cases [4], community involvement and strong stakeholder collaboration are essential [77]. The key components that drive the success of AFP surveillance are described in Table 1. These key elements include conducting house-to-house surveys, especially in remote areas, to detect possible AFP cases, establishing effective communication channels for community reporting, ensuring timely collection and of analysis laboratory samples, and collaborating with non-governmental organizations (NGOs) and the surrounding community in the observation and monitoring process. The key elements also include adherence to certification standards, public awareness campaigns, robust data management systems, and adequate resource allocation to address the challenges posed by AFP effectively.

**Table 1:** Essential components for sensitive acute flaccid paralysis surveillance

Components	Description
Community engagement	Emphasize the role of educated and selected community members in seeking stool specimens, increasing awareness of AFP, and reporting clinical cases to district health offices
Active case searching	House to house surveys should be conducted to locate potential AFP patients especially in areas that are comparatively secluded
Vaccination coverage	To achieve high vaccination coverage program to eliminate polio and other relevant disease that causes paralysis
Timely reporting	Develop channels for reporting, ensure the volunteers and health personnel in the community get to report any case of AFP to the appropriate persons
Sample transportation	Ensure that the specimens are collected in a proper manner and transported to appropriate laboratories at the right time
Laboratory capacity	Make sure that laboratories have all it takes to analyze the fecal samples appropriately and find out the root cause of AFP
Collaborative support	Involve NGOs and the surrounding community in multiple observation practices to guarantee inclusiveness and backing
Follow-up examination	Examine patients to evaluate the extent of the residual paralysis a month after the manifestation of paralysis symptoms
Certification standards	Adhere to certification standards by way of the surveillance system complies with the wanted quality and performance standards
Training	Sensitize and support leaders in communities and health related sectors for first responder surveillance in AFP
Public awareness campaigns	Conduct information, education, and communication (IEC) activities regarding the awareness of AFP surveillance and ways on how to report suspicious cases
Data management systems	Optimum information systems in the form of databases should be used to monitor and document cases of AFP to increase efficiency in record keep and reporting
Resource allocation	It is vital that sufficient funds are provided for surveillance, includes human resources, modes of transport, and reagents for laboratory tests
Evaluation and monitoring	Ensure that the surveillance system is periodically checked and audited to know the challenges faced in the area

## AFP Surveillance Performance Indicators

AFP surveillance performance is consistently assessed using established surveillance quality metrics used as benchmark for effectiveness of polio eradication operations [78]. The indicators aid in clarifying the validity of surveillance processes including, data collecting, processing, and presentation [79].

**Fig. 6:**
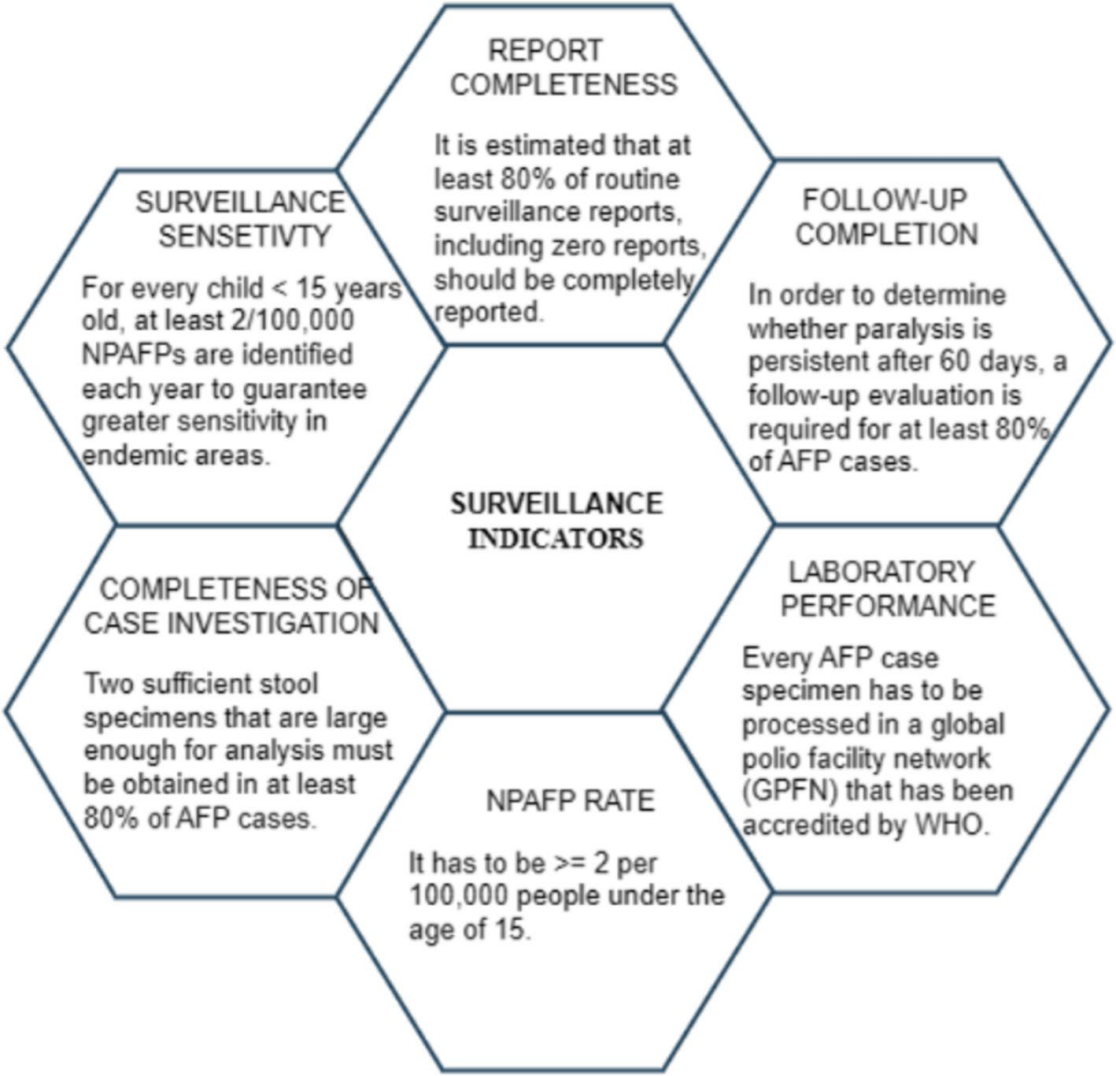
Quality indicators used to determine the performance of acute flaccid paralysis surveillance. NPAFP, non-polio acute flaccid paralysis; WHO, world health organization

According to GPEI, there are several surveillance indicators that include completeness of the case investigation, report completeness, efficiency, comprehensiveness of data, turnaround time for reporting, stool sample trasportation to laboratory, and the timeliness of detection [80–82] (see Fig. 6). In addition to indicators mentioned above, there exists two key performance indicators used to assess the quality of AFP surveillance. These are collection of adequate stool specimens from AFP patients (with a target of ≥ 80%) [83, 84] and the non-polio AFP (NPAFP) rate (defined as ≥ 2 per 100,000 people under the age of 15 as sufficiently sensitive to detect circulating PV) [1, 47, 85]. For AFP surveillance to be sensitive, all samples must be processed at a laboratory authorized by the WHO, and > 90% of monthly reports must be received with a minimum of 80% arrival rate. Health authorities and designated focal persons can utilize these indicators to make sure they know what has to be improved, strengthen case identification, and make sure that they collect high-quality surveillance data that will aid in the global fight against polio.

## Types of AFP Surveillance

**Fig. 7:**
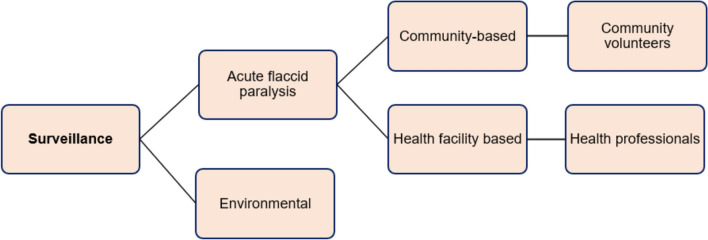
Types of polio surveillance

Polio surveillance can be categorized into two types as environmental surveillance and acute flaccid paralysis surveillance. AFP surveillance is further classified into two types to detect the transmission of PV, namely health facility-based and community-based AFP surveillance [86] (see Fig. 7). Health facility-based surveillance is a way of conducting surveillance of AFP in the health facilities to report cases of paralysis using skilled personnel, who mostly work in health facilities such as hospitals and clinics. However, in CBS, communities are expected to ascertain symptom of AFP based on simple community case definition. Thus, when a child or an adult is paralyzed, close relatives and community volunteers are supposed to inform the village health worker or the focal person assigned in the respective village [24]. The focal persons in turn pass any suspected case information to the next health authorities for further investigation. Thus, this decentralized approach guarantees that isolated territories also be covered by community. Furthermore, CBS approach of AFP is taken as a crucial means for timely case intervention.

## Community-Based AFP Surveillance

**Fig. 8:**
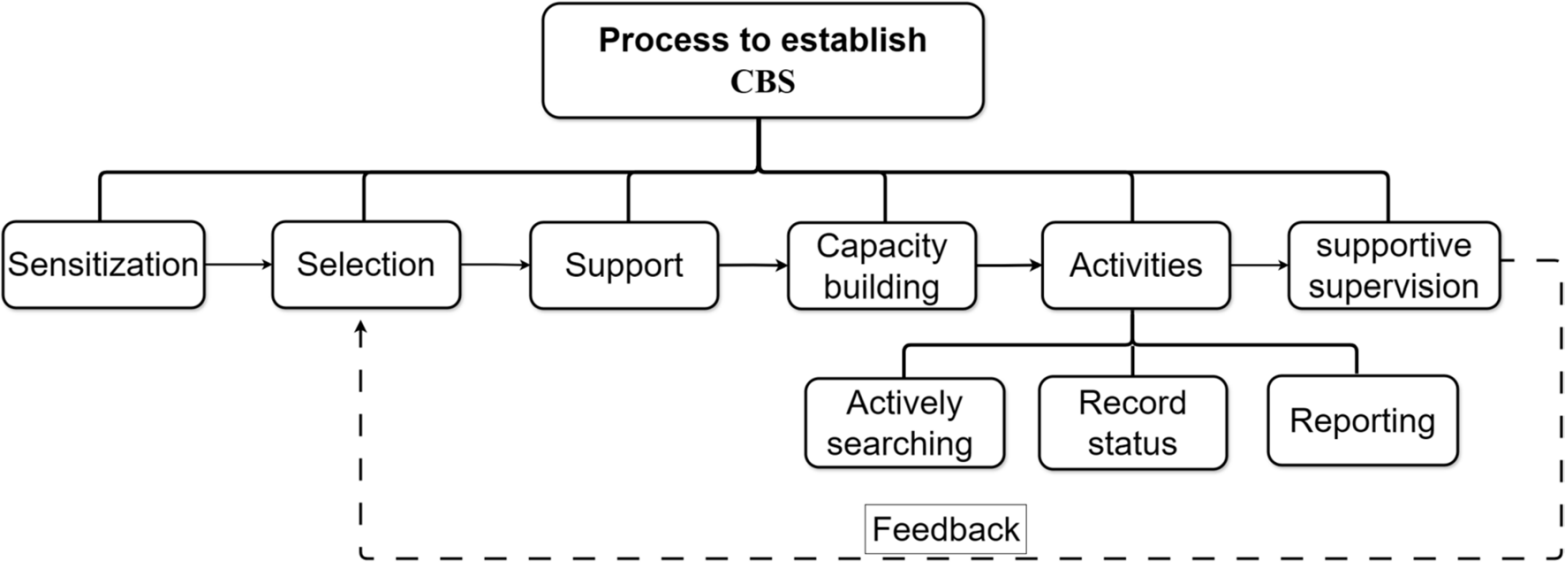
The process to establish effective community-based AFP surveillance

CBS for AFP is an essential intervention that aims at early identification and notification of potential cases and is playing an important role in polio control efforts [32]. This strategy leverages the involvement of community members and local health workers in actively searching cases and reporting possible signs of paralysis caused by PV. The CBS way of intervention is crucial as it enables early detection of the disease that helps to control the spread and take response action in shortest period of time possible. In CBS, community participation is supported by different activities including, awareness creation campaigns on AFP case definition, empowering communities, building trust and changing attitudes using responsible persons for reporting [87, 88]. This approach significantly enhances scope of surveillance activities, reaching polio hot spots and remote or underserved areas where limitation in healthcare infrastructure exists [89, 90]. A rigorous process is required to establish CBS for an effective AFP monitoring system [39]. The establishment process begins with raising awareness to find and identify a potential volunteer from the community members. From among the identified volunteers, some of the potential and acceptable volunteers are chosen, and any assistance in supporting the surveillance process, such as information documenting tools are provided to the selected personnel. The selected CVs then needs support for capacity-building in surveillance-related tasks like record status, actively searching, and reporting. Moreover, supervision of the functionality of CVs, provision of extra and refresher training to select new CVs and improve the sensitivity of surveillance performed by existing volunteers is needed (see Fig. 8).

**Fig. 9:**
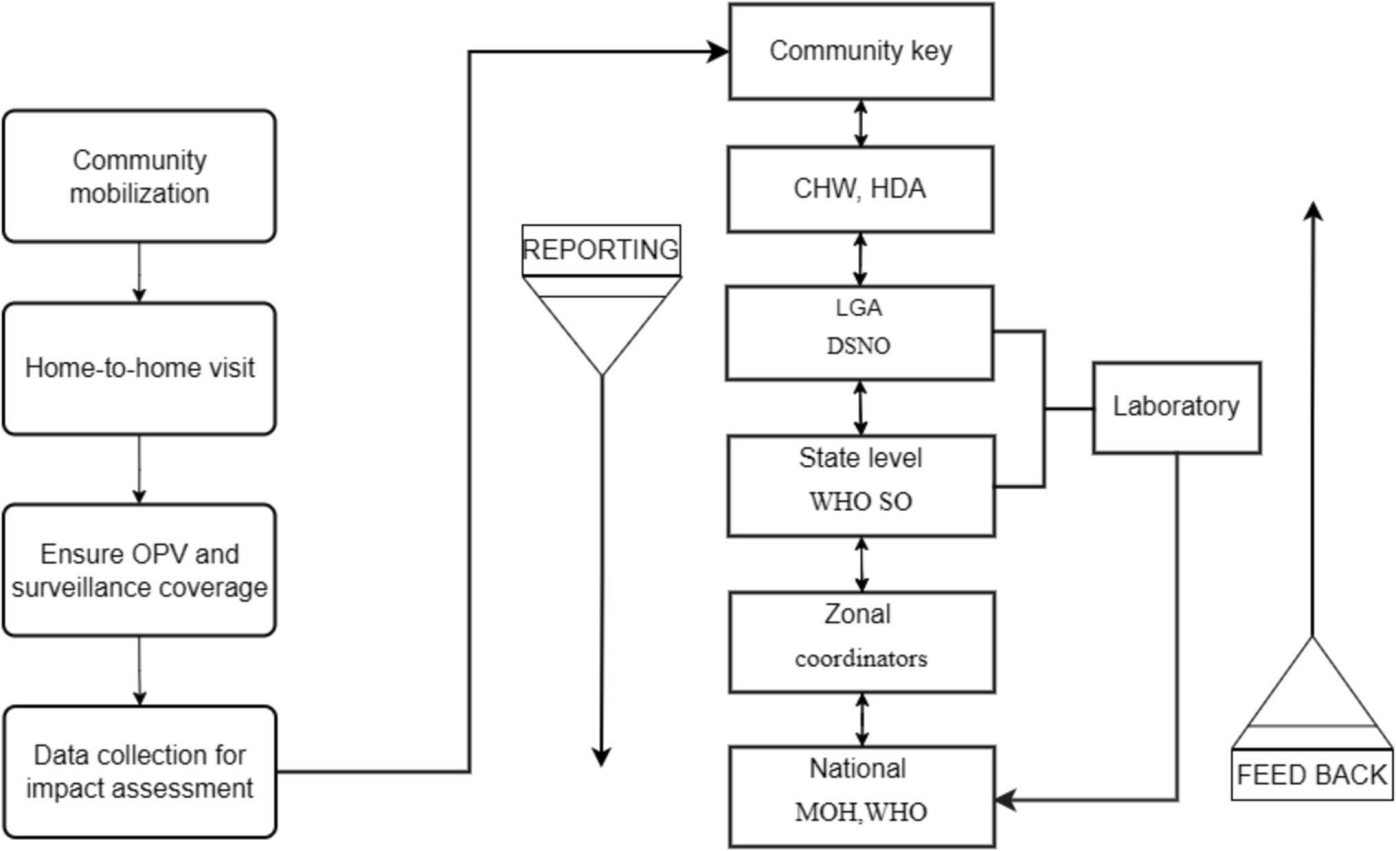
The data flow of CBS of AFP mapping the process from the collection of data by community key informants during home visits to its reporting at the national level, including the WHO-accredited laboratories. *LGA* Local Government Area, *WHO SO* World Health Organization Surveillance Officer, *MOH* Ministry of Health

The CBS system has a structure for flow of information [38]. In Fig. 9, a data flow diagram (DFD) that shows all the activities involved in the transfer of information from the community to health facility and vice versa are well described. A coherent data flow approach of CBS of AFP necessitates timely identification of the existing cases, their proper diagnosis, and aids to give adequate feedback for further dissemination and protection of health of population. During the surveillance activity, community key informants are expected to report all necessary informations needed including name, gender, age (because clinical manifestations can vary by age) [91], location of residence, the name of the informant, date of notification, date of investigation, date of sample collection, date that the specimen transported to laboratory, and other information [56, 92]. This comprehensive data flow ensures accurate tracking and timely response, enhancing the effectiveness of surveillance efforts and aiding in the early detection and control of disease outbreaks.

## Improvements Brought About by CBS of AFP for Polio Eradication

The purpose of CBS is to guarantee that disease cases that are within the community are not missed so that the officials monitoring disease surveillance system get information early and provide rapid response [56]. Prior to the widespread adoption of CBS, virtually little was done on AFP cases and the disease's onsets were not promptly reported. However, when the local community is engaged as a foundational source of solutions for public health, a significant shift began to happen. The initiative considerably contributed in the eradication of disease using heightened community involvement.

The implementation of CBS for AFP has led to the growth and utilization of the community, greatly enhancing public health efforts and ensuring that no cases are left undetected or unnoticed. Among the noteworthy improvements are community activities like early detection and prompt reporting, which enable prompt investigation and case interventions [93]. CBS strategy increased number of reporting AFP case, particularly in remote and underserved area where health facilities are limited. Moreover, the involvement of communities in AFP surveillance has heightened public awareness on the importance of early reporting and encouraged greater participation in disease control efforts. The quality of data collected on AFP case were also improved. Furthermore, the integration of CBS into broader public health systems has strengthened overall health infrastructure. By building local capacity and training community members, health systems have become more resilient and better equipped to handle AFP and other health challenges. Improvements brought in some countries discussed as follows.

For instance, in the use case of Ethiopia, core group polio project (CGPP) has been operational focusing on polio eradication through financial support from USAID since 1999. Since then, CGPP and other initiatives created successful community-based intervention and social mobilization to counter epidemic disease. Reports from CGPP suggests that CBS for AFP has enhanced case detection and reporting in its implementation sites [38]. Due to AFP monitoring methods of CGPP through community participation, the surveillance activity improved by 30% in the past two decades [44]. This approach helps with the timely identification and management of potential polio cases by raising community awareness and promoting stronger ties between the community and the health system. Significant enhancements of the use of CBS in detection of AFP case have also been documented in Nigeria in recent years. CBS has been vital in eradicating the polio threat and maintaining nation’s polio free status [39]. It was achieved through deployment CBS in high-risk areas in Nigeria [57]. This program has been crucial in quickly identifying and treating instances of polio, which helped to eradicate the WPV.

With CBS being implemented across the world, outbreak control measures have improved and the world is now one step closer to being polio-free. In addition to achievements seen due to community participation, CBS of AFP is further aided by deployment of advanced technological tools for detection and reporting mechanism. The tools enhanced the surveillance system by automating case detection and predicting outbreaks [94].

## Technological Advancements in AFP Surveillance

Surveillance operations in the monitoring of public health have seen significant enhancement due to advancement in technology as these resulted in better tools that enhance data gathering and reporting. Technological tools in the surveillance of AFP improved the capability and efficiency of the system. Some of the improvements include the use of digital reporting platforms, mobile applications and geographical information systems that possess data acquiring and transmitting capabilities at real time. The tools supported to capture and report data by replacing the conventional paper-based systems with efficient real-time entries leading to quicker investigation [95–97]. The tools further facilitate surveillance activities via remote supervision with real-time data exchange to improve the provision of intervention and feedback to CVs. These tools are also convenient to map AFP cases, the VDPV outbreak incidences, and vaccination programs to quickly find regions with low vaccination [98]. Automated reporting technology enhances the speed in which the data is transferred to the central databases thereby speeding the process of analysis and response. Furthermore, these technologies facilitate the visualization of data through interactive approaches make easier for public health officers to identify trends and attentiveness, track the disease spread and manage resources. Additionally, some tools have integrated warning systems that helps to give alert on any disease detection. In addition to that, the tools safeguard security and privacy of the data in the health facility whereby sensitive patient data is well protected while at the same time enabling the sharing of the data for analysis. Therefore, through the use of tools mostly installed on mobile phones of CVs, health authorities can advance the AFP surveillance activities such as case identification, investigation and response that improve the general health of the public, leading to eradication of polio. Table 2 presents key digital technology tools designed to enhance CBS for AFP. It includes deep learning methods used in Ethiopia to process images of suspected AFP cases, aiding in early detection and intervention [44]. Furthermore, AVADAR is implemented as a real-time reporting tool that allows community members to report AFP cases and sends immediate notifications to supervisors [99]. Abridged electronic surveillance (eSurve) checklists are used for tracking and reporting surveillance visits [95], while the ODK supports timely data collection and improved case geo-encoding [43]. Integrated supportive supervision (ISS) tracks vaccine-preventable diseases and immunizations by logging supervisory visits and monitoring coverage [100]. Moreover, the electronic surveillance system for the early notification of community-based epidemics (ESSENCE) offers a web-based platform for monitoring public health indicators and tracking outbreaks through spatial mapping and custom alerts [95]. Collectively, these tools enhance the efficiency, accuracy, and responsiveness of AFP surveillance efforts.

**Table 2:** Summary of the digital technology tools implemented to improve CBS system

Innovation	Description	Significance	Tool	References
Deep learning for improved CBS AFP in Ethiopia	Deep learning method for identifying AFP cases from images of suspected child collected by KCIs	Aids in bridging the gap between detection of CVs and response of health facilities, guarantees early intervention and safely stores data	Mobile phone	[44] Ayana et al
Auto-Visual AFP Detection and Reporting (AVADAR)	A reporting and monitoring tool for CBS that makes it possible for community members to identify and report cases of AFP	Notifies designated supervisors any suspected AFP cases in real time and at the specified location. Its 30-s AFP case video aids in triggering searches	Mobile phone	[99] Shuaib et al
Abridged electronic surveillance (eSurve) checklist	Active surveillance visits monitoring and reporting system in real time	Tracks surveillance coverage, time, location and record demographic data on visits	Mobile phone	[101] Edukugho et al
Open data kit (ODK)	Its open-source tool, to collect, transfer and store data	It’s important for timely data availability, enhanced AFP case geo-encoding, and prompt medical measures	Mobile phone	[43] Malaghemi et al
ISS (Integrated supportive supervision)	A real-time system for tracking and reporting on the occurrence of vaccine-preventable diseases and important immunizations	Logs supervisory visit time, location, and data. Shows patterns over time and date. and also monitors visit coverages	Mobile phone	[100] Sadiq Umar et al
ESSENCE (electronic surveillance system for the early notification of community-based epidemics)	A secured web-based application, healthcare providers can monitor public health indicators that are crucial for tracking, identifying outbreaks and other significant events	Spatial mapping, remote data collecting, event communications, user-defined alert messages, and custom querying	Mobile phone	[95] Burkom et al

Deep learning has been shown to be effective in detecting AFP cases by using CBS [44] with vision transformer architectures improving the accuracy of AFP detection from images collected by community informants. However, its evaluation highlighted limitations, including data quality issues stemming from insufficient training for community volunteers in capturing high-quality images. Additionally, reliance on smartphones and internet connectivity presents challenges in remote areas. While the potential for scalability across various socio-economic and geographic contexts of the deep learning approach is significant, it requires careful consideration of local healthcare infrastructure, cultural attitudes towards disease reporting, and resource availability. To enhance scalability, it is crucial to develop adaptable frameworks tailored to the unique needs of diverse communities. This includes providing adequate training for volunteers, ensuring access to reliable technology, and fostering community engagement through awareness campaigns.

The SMS-based smartphone application Auto-Visual AFP Detection and Reporting (AVADAR) offers significant advantages for detecting and reporting AFP cases [99]. Designed to enhance AFP surveillance, AVADAR improves sensitivity by enabling quick case detection and reporting through a user-friendly interface, thereby boosting the efficiency of the surveillance network. It raises awareness among community informants and healthcare workers with educational videos, leading to better case identification. The application facilitates effective communication by allowing instant notifications of AFP cases, with minimal information sent directly to authorities for investigation. Its geolocation capabilities and feedback loop mechanisms enhance accurate case tracking and follow-up. Pilot studies indicated notable improvements in AFP detection and reporting. However, challenges like poor network connectivity and reliance on smartphones may exclude populations without access or digital literacy, creating disparities. Despite these limitations, AVADAR can adapt to various public health priorities and engage community members effectively. Successful scalability will depend on addressing local cultural and socio-economic factors and incorporating community feedback.

Abridged electronic surveillance (eSurve) checklist was implemented to improve AFP surveillance during the COVID-19 epidemic in Nigeria, significantly enhancing data collecting efficiency and real-time reporting [101]. However, its effectiveness was challenged by adaptation difficulties for personnel used to the longer original checklist, which could lead to inconsistencies in data collection. Furthermore, different states implementations led to diverse results in terms of AFP case detection. Despite these drawbacks, the adaptability of the checklist suggests that it could be scaled for use in other health surveillance situations, especially those with limited resources.

In 2017, South Sudan implemented ODK mobile data collection technology to strengthen its AFP surveillance system, with the goal of increasing the efficiency and accuracy of data gathering, which is crucial for monitoring and responding to polio outbreaks [43]. This deployment enabled health personnel to collect real-time data, including the geocoded locations of AFP cases. However, the assessment of ODK's efficacy has mostly relied on restricted case studies and anecdotal data, suggesting that more thorough evaluations like randomized controlled trials are required to completely comprehend its influence. Among its drawbacks were security issues and inadequate internet access in rural areas. Furthermore, the implementation underlined the importance of providing proper training for health workers in order to ensure high-quality data gathering. Scalability of ODK requires careful consideration, as regions with strong infrastructure and trained personnel can significantly improve health surveillance and intervention efforts, whereas low-resource settings may face challenges such as limited access to technology and varying levels of digital literacy. Thus, it is essential for success to modify implementation tactics to suit local conditions. Overall, the case of ODK implementation in South Sudan demonstrates both the potential and challenges of using mobile technology for health surveillance, emphasizing the importance of ongoing evaluations, addressing limitations, and strategic planning for scalability in order to maximize its impact in diverse settings.

Another important case study investigated the influence of ISS on health worker performance in Zambia, focusing on issues in surveillance and immunization [100]. The study evaluated the percentage point difference in performance metrics between first and recent visits, as well as the frequency of ISS visits. The results showed that by improving surveillance and immunization efforts through active case searches, on-the-job training, and mentorship, ISS improved health worker performance. Issues with data quality, the requirement for sufficient training, and dependence on dependable internet connectivity were among the drawbacks. Since effectiveness is greatly influenced by local government, community involvement, and resource availability, scalability of these technologies across many settings is crucial.

ESSENCE is a web-based application designed to effectively monitor health threats and enhance communication among healthcare providers [95]. However, its efficacy is dependent on the quality of the data, relying only on counts, may miss important low-count scenarios, and relying on zip codes may limit geographic precision as the implementation area does not have zip code for each house. Additionally, user expertise is necessary for effective data interpretation, which can be challenging in understaffed settings. Despite these challenges, ESSENCE’s modular architecture allows for customization based on local health concerns, enhancing its relevance. Integration with existing health information systems is also crucial for broader implementation.

## Challenges in CBS of AFP

Community-based surveillance for AFP is considered as a key for early detection and timely reporting. However, it has limitations that hinder its functionality [102]. Some of them include inadequate access to healthcare facilities, lack of community awareness and engagement, political instability, the community's nomadic lifestyle, cultural and linguistic differences, inadequate infrastructure, low communication networks, volunteer demotivation, the distance between homes and health facilities, lack of information on the need for repeated vaccinations [103], and a lack of logistics for conducting surveillance are factors affecting CBS [14]. Some of key challenges are discussed as follows.

### Lack of Awareness and Community Engagement

Effective surveillance relies on enlightened and active participation of community members and community volunteers. Lack of knowledge on signs and manifestation of AFP [104], leads to under-reporting [96], exclusion of other community risks related to novel strains of infectious and viral disease, vaccine refusal [105, 106], inconsistency in qualitative data collection [35], delayed reporting, and inadequate data interpretation, analysis, and usage that further weakens the AFP surveillance systems. Furthermore, the failure of CVs to report and record missed or incomplete data at the community level, as well as to report zero doses during immunization activities, leads to the emergence of gaps. Lack of knowledge on usage of reporting tools such as mobile phone applications are also considered as additional limiting factors [37].

Cultural beliefs also impact reporting behavior, as some cultures associate paralysis with witchcraft or avoid discussing it altogether. Addressing these issues requires combating prejudice and, crucially, raising awareness about the significance of paralysis for public health. A healthy relationship between the health workers and the community through active community engagement minimizes the risk of challenges [34].

In rural Ethiopia, implementing a CBS system for AFP encountered significant obstacles, primarily due to low community awareness about the disease and its symptoms [44]. Many residents lacked understanding of the importance of timely reporting, resulting in underreporting and delays in detecting potential AFP cases. Cultural beliefs and misconceptions further hampered community engagement, limiting opportunities for intervention and increasing the risk of poliovirus transmission. To address these challenges, targeted awareness campaigns were introduced, leveraging the influence of local leaders and health workers to educate communities about AFP and the pivotal role of KCIs in disease surveillance [4]. These efforts led to substantial improvements in community engagement and reporting rates, significantly enhancing the effectiveness of the CBS system and contributing to improved public health outcomes. This shows the critical role of community awareness in the success of surveillance initiatives, especially in low-resource settings.

Similarly, in Pakistan, the implementation of polio vaccination programs has faced resistance due to widespread misconceptions linking the vaccine to infertility [31]. These fears, deeply rooted in cultural and religious beliefs, have led many individuals to refuse vaccination. Community decisions are strongly influenced by these perceptions, posing a significant barrier to achieving immunization goals. Addressing these challenges requires culturally sensitive strategies, including engaging trusted community and religious leaders to dispel myths, build trust, and promote the safety and benefits of polio vaccines.

### Security Issues Due to Political Instability

CBS is significantly hindered by security concerns. Surveillance, primarily conducted by community health workers (CHWs) and CVs in remote and inaccessible areas, places these workers at risk due to conflicts and instability in these regions. The safety of surveillance personnel is jeopardized by the volatile conditions they encounter while performing their duties. Their safety is at risk, as a result, they cannot get close to patients because of restricted mobility that limits the chance of case reporting, or even get the correct number of reported AFP cases. Instability can hinder access to remote areas and disrupt telecommunication systems, such as mobile phones and radios, which can lead to faults and make timely reporting challenging. Consequently, limited access also weakens the effectiveness of response and investigation efforts [40].

For example, in Nigeria, Boko Haram attacks have led to violence against polio surveillance workers and disruptions in polio immunization campaigns, resulting in limited access to healthcare and vaccination services [107]. In Pakistan, millions of people have been internally displaced due to the conflict between the military and rebels operating from polio-endemic regions. This displacement has contributed to the widespread transmission of WPV and has disrupted surveillance activities [108]. The current poliovirus outbreak in Gaza, in 2024, which endangers many children, is largely due to political instability. This instability has resulted in irregular immunization coverage and disrupted surveillance efforts [109]. The PV outbreak in Gaza in 2024 is significantly influenced by the region's ongoing political instability. The conflict and unstable conditions have led to disruptions in routine healthcare services, including regular immunization programs. As a result, children in Gaza have missed crucial vaccinations that protect against poliovirus. Additionally, the instability has hindered efforts to monitor and control the spread of the virus, exacerbating the outbreak and putting more children at risk.

### Nomadic Lifestyle of the Community

Due to the high population mobility and the communities’ frequent movement across various regions within the targeted groups where CBS is implemented, it is nearly impossible to carryout surveillance consistently [44]. Their mobility impacts the reporting and monitoring schedules. These mobile community are difficult to access by health workers, which hinders the identification of new cases of AFP. Follow-up efforts are further complicated by frequent changes in settlement patterns. Addressing these challenges requires tailored approaches, active community participation, and collaboration with local leaders to ensure effective AFP surveillance among mobile populations.

In regions with high population movement, particularly in remote and conflict-affected areas, CBS was implemented as an innovative strategy to enhance disease surveillance and vaccination efforts, particularly in Kenya and Somalia [32]. Recognizing the limitations of traditional health interventions, local volunteers were used as community mobilizers (CMs) who understood their communities and effectively monitored health issues. Furthermore, to facilitate collaboration among health systems, cross-border health committees were established that addressed not only polio but also other outbreaks like cholera, ensuring mobile populations received essential services. Additionally, targeted immunization outreach provided transportation for health staff to reach high-risk groups, improving access to vaccinations. CBS also tailored communication strategies, with CMs delivering culturally relevant messaging to foster trust and encourage participation in vaccination programs. Through these approaches, the CBS system for AFP successfully navigated the challenges of the population movement, demonstrating the effectiveness of community engagement and adaptive strategies in public health initiatives.

### Emergence of New Pandemics

During new pandemics, resources, personnel, and attention shifts to contain the outbreak of the disease. Thus, CBS for AFP receives less attention that results in poor surveillance activities and reporting. It contradicts the concept of CBS because health workers involved in it will be moved to responds to other tasks related to the new pandemic. In other words, their absence or reduced capacity delays the diagnosis and reporting of cases like AFP. The outbreak of emerging infectious diseases caused by many reasons also aggravates the distortion on reporting and recording of AFP [110]. Following the emergency epidemic disease outbreaks, the CVs and even budgets planned for the AFP surveillance purpose shifts to the new pandemic which in turn limits the AFP case detection. As a result, a lot of AFP symptoms are left unnoticed or not be reported.

For instance, during the COVID-19 pandemic, AFP surveillance activities experienced a significant decline, reaching their lowest levels in a decade [111]. The pandemic's overwhelming impact on healthcare systems diverted resources and attention away from routine surveillance and immunization programs, including those crucial for monitoring AFP cases. Lockdowns, movement restrictions, and the reallocation of healthcare personnel to COVID-19 response efforts further disrupted AFP surveillance. As a result, the ability to detect and respond to new cases was severely compromised, increasing the risk of undetected transmission of poliovirus and other related diseases. This decline highlighted the vulnerability of disease surveillance systems during global health crises and underscored the need for resilient and adaptable public health strategies.

### Resource Constraints

Inadequate resources allocated for AFP surveillance operations are viewed as limitations. As one tool for surveillance activity, financial support is used to pay for field expenses incurred during data collection (activity costs), to supply tools that facilitate the reporting process, to build capacity (by providing training), to cover transportation costs, to provide technical support, and to encourage CVs through incentives [58]. However, the termination of financial support highly challenges AFP surveillance.

In low-resource areas, surveillance activities are significantly hindered due to inadequate infrastructure and limited resources [14]. For instance, poor roads and infrastructure make home visits challenging, while a lack of transportation further exacerbates these difficulties. Even when home visits are conducted, timely communication with designated focal persons often proves difficult, further impeding surveillance efforts. To address these challenges, it is essential to engage stakeholders to invest in and support surveillance activities. This includes improving infrastructure, providing reliable transportation, and establishing effective communication systems to ensure timely reporting and coordination. Such measures will strengthen surveillance efforts and enhance their reach in low-resource settings.

## Outlooks

In underprivileged communities and locations typically classified as AFP-risky areas, involving the general-public in the reporting of cases to the formal health system increases the sensitivity of surveillance. Efforts need to be made to increase the sensitivity of AFP surveillance system beyond what it now is, by considering the challenges that currently exist and attempting to find a better solution.

CBS depends on well-functioning health systems, these systems become exhausted when a new pandemic hits. Thus, incorporating an evolutionary approach, collaborating with the community, and maintaining illness awareness in times of potential pandemics are imperative in this situation. Furthermore, to combat under-reporting, it is necessary to include the community, run educational efforts, improve reporting channels, and make sure that AFP is identified as soon as feasible.

Effective surveillance is further aided by activities like building relationships with local leaders and giving health workers' safety-first priority. Continuous training and feedback also serve to monitor the CHWs' motivation to work on surveillance tasks. The foundation of effective AFP monitoring at the community level is essentially the creation of trust through community mobilization and promotion.

The CBS effort is further improved by involving stakeholders from various sectors, including academia, business, NGOs, community leaders, civil society, and other pertinent parties in a cooperative and coordinated effort to advance surveillance techniques and ensure adequate readiness for responding to outbreaks [112]. Putting in place a unique surveillance system that includes leveraging powerful organizations to quickly report incidents and concentrating more on cross-border integrations in regions where the virus spreads is crucial.

Moreover, deployment of technological tools for AFP surveillance in order to keep track, report, and thoroughly record all relevant data in real time aids the field. The introduction of artificial intelligence and machine learning model further improves the surveillance system allowing early detection and intervention of outbreaks [94].

Generally, efforts to strengthen CBS of AFP should prioritize immunization campaigns, training, community engagement, funding for regular surveillance visits, and the systematic inclusion of additional community informants, with technology serving as a key tool. To fully eradicate polio and strengthen CBS, all stakeholders must pursue innovative initiatives, collaborate with local leaders, and prioritize the safety of healthcare workers.

## Conclusion

The progress of CBS for AFP has been thoroughly reviewed, showcasing its vital role in advancing poliovirus eradication efforts. CBS enhances community knowledge, enabling early detection and timely reporting of suspected AFP cases. However, challenges such as security concerns, delayed reporting, low community awareness, emerging pandemics, and limited use of innovative technologies persist. Addressing these issues requires attention to organizational structures, healthcare access, and cultural contexts.

Key recommendations include raising community awareness to improve understanding of AFP case definitions and expanding the use of technology for early reporting. Collaboration with local authorities and health professionals is essential to enhance community engagement and streamline detection and reporting processes. Investments in AI-powered tools and mobile health applications are recommended to improve surveillance efficiency, facilitate real-time reporting, and support outbreak prediction. Training healthcare workers and community volunteers in data collection and response protocols is critical for preparedness. Strengthening partnerships with academic institutions, NGOs, and community organizations can enhance resource sharing and surveillance effectiveness. Robust data analysis methods should be developed to guide public health decisions, optimize resource allocation, and adapt strategies based on AFP trends. Establishing mechanisms for monitoring and evaluating CBS activities with regular feedback loops will ensure the system remains responsive and adaptive.

This paper highlights the vital role of CBS in improving disease detection and response, extending its impact beyond polio eradication to other diseases such as malaria, tuberculosis, neonatal tetanus, anthrax, and rabies. It offers a framework for integrating technology and community involvement to strengthen public health strategies and build robust health systems. These insights contribute to safeguarding community health and advancing global disease prevention efforts.

## Data Availability

No datasets were generated or analysed during the current study.
